# Relationship between hypertension and non-obstructive coronary artery disease in chronic coronary syndrome (the NORIC registry)

**DOI:** 10.1371/journal.pone.0262290

**Published:** 2022-01-21

**Authors:** Caroline A. Berge, Ingeborg Eskerud, Elise B. Almeland, Terje H. Larsen, Eva R. Pedersen, Svein Rotevatn, Mai Tone Lønnebakken

**Affiliations:** 1 Department of Heart Disease, Haukeland University Hospital, Bergen, Norway; 2 Department of Clinical Science, University of Bergen, Bergen, Norway; 3 Department of Biomedicine, University of Bergen, Bergen, Norway; University of Bologna, ITALY

## Abstract

**Background:**

The burden of non-obstructive coronary artery disease (CAD) in the society is high, and there is currently limited evidence-based recommendation for risk stratification and treatment. Previous studies have demonstrated an association between increasing extent of non-obstructive CAD and cardiovascular events. Whether hypertension, a modifiable cardiovascular risk factor, is associated with extensive non-obstructive CAD in patients with symptomatic chronic coronary syndrome (CCS) remains unclear.

**Methods:**

We included 1138 patients (mean age 62±11 years, 48% women) with symptomatic CCS and non-obstructive CAD (1–49% lumen diameter reduction) by coronary computed tomography angiography (CCTA) from the Norwegian Registry for Invasive Cardiology (NORIC). The extent of non-obstructive CAD was assessed as coronary artery segment involvement score (SIS), and extensive non-obstructive CAD was adjudicated when SIS >4. Hypertension was defined as known hypertension or use of antihypertensive medication.

**Results:**

Hypertension was found in 45% of patients. Hypertensive patients were older, with a higher SIS, calcium score, and prevalence of comorbidities and statin therapy compared to the normotensive (all p<0.05). There was no difference in the prevalence of hypertension between sexes. Univariable analysis revealed a significant association between hypertension and non-obstructive CAD. In multivariable analysis, hypertension remained associated with extensive non-obstructive CAD, independent of sex, age, smoking, diabetes, statin treatment, obesity and calcium score (OR 1.85, 95% CI [1.22–2.80], p = 0.004).

**Conclusion:**

In symptomatic CCS, hypertension was associated with extensive non-obstructive CAD by CCTA. Whether hypertension may be a new treatment target in symptomatic non-obstructive CAD needs to be explored in future studies.

**Clinical trial registration:**

ClinicalTrials.gov: Identifier NCT 04009421.

## Introduction

Non-obstructive coronary artery disease (CAD) is commonly detected by coronary computed tomography angiography (CCTA) in patients with chronic coronary syndrome (CCS) [1]. Even though non-obstructive CAD is associated with an adverse prognosis, recommendations for risk stratification and management are still inadequate [2–5]. High coronary calcium score as well as extent of coronary artery atherosclerosis by CCTA have previously been identified as important risk markers [6, 7]. In particular, extensive non-obstructive CAD has been associated with the highest cardiovascular (CV) event rate [7]. According to previous research, statin therapy seems to be beneficial, particularly in high-risk individuals, while aspirin may be harmful in subgroups of patients with low risk [8–10]. Recently, a dedicated risk score model for non-obstructive CAD combining clinical and angiographic characteristics was suggested to identify high-risk individuals [11].

Hypertension has been established as a modifiable CV risk factor through several studies demonstrating a significant CV risk reduction by antihypertensive treatment [12, 13]. The burden of hypertension in the world remains high and is expected to increase further in the upcoming years [14]. It has been demonstrated that CAD is more prevalent among hypertensive subjects and that the risk of CAD increases with increasing systolic blood pressure (BP) [15]. Hypertensive mediated organ damage like left ventricular hypertrophy and increased arterial stiffness have also been associated with myocardial ischemia in symptomatic non-obstructive CAD [16, 17].

Even though current guidelines recommend initiation of antihypertensive drug treatment in patients with high normal BP or hypertension with established CV disease or subclinical atherosclerosis detected by CV imaging, recommendations for antihypertensive treatment in patients with CCS and non-obstructive CAD remain unclear [12, 13, 18, 19]. Hence, the aim of this study was to investigate the independent relationship between hypertension and extent of non-obstructive CAD by CCTA in patients with CCS from a large clinical registry.

## Methods

### Study population and data source

We conducted a cross-sectional registry-based study using the Norwegian Registry of Invasive Cardiology (NORIC), a national quality control registry. The study includes a total of 1138 individuals electively referred to CCTA due to suspected CAD and stable chest pain or dyspnoea. The patients were diagnosed with atherosclerotic plaques in the coronary arteries with a lumen diameter reduction between 1–49% by CCTA at the Department of Heart Disease, Haukeland University Hospital, Bergen, Norway, between January 2016 and September 2019. Patients with smooth coronary arteries, obstructive CAD, previous myocardial infarction, heart surgery and percutaneous coronary interventions were excluded. Clinical and CCTA characteristics of the study population were extracted from the registry. The study was approved by the South-East Regional Ethical Committee for Medical and Health Research Ethics in Norway (reference number REK 2017/1573). The participants’ records and information were de-identified; hence the requirement of informed consent from participants was waived. The study was also registered at ClinicalTrials.gov with identification number NCT04009421.

### Cardiovascular risk factors

Patient information on demographics, CV risk factors and CV disease were reported on a standardized questionnaire and quality controlled against patients’ records by the registry staff. Systemic arterial hypertension was defined as known hypertension or use of antihypertensive medication. Diabetes mellitus was defined as known diabetes or on antidiabetic treatment. Body mass index (BMI) was calculated as body weight divided by height in meters squared, and obesity was defined as BMI≥30kg/m^2^. Smoking was classified as current or former smokers. GFR was estimated using the CKD-EPI equation [20]. Clustering of CV risk factors was considered present if at least 3 CV risk factors were detected in the same individual.

### Coronary CT angiography

CCTA was performed on clinical indication by dual source scanners (Somatom Definition Flash 2x 128-slice or Somatom Force 2 x 192-slice, Siemens, Germany) with electrocardiographic gated acquisition following current guidelines [21]. In patients with heart rate > 60 beats per minute, metoprolol 1 mg/ml (maximum 20 mg) was administered intravenously until heart rate was < 60 beats per minute. An initial non-contrast enhanced scan was performed first to determine coronary calcium score. Then all patients received non-ionic contrast intravenously as iomeprol 400mg I/ml (Iomeron, Bracco, Milan, Italy) according to body weight. In addition, 0.4 mg nitroglycerine was administered sublingual prior to CCTA in order to improve image quality.

All CCTA images were analysed by experienced readers using a dedicated analysis software, SyngoVia (Siemens, Germany). Coronary artery calcium score was reported as area-density (Agatston score) in HU. Non-obstructive disease was visually identified as a stenosis with 1–49% lumen diameter reduction in any segment of the coronary arteries using the modified 20-segment American Heart Association classification [22]. All patients with at least 1 stenosis of ≥50% were excluded [22]. Segment involvement score (SIS) was calculated as the number of segments with 1–49% stenosis, and extensive non-obstructive CAD was defined as SIS>4 [7, 22]. Furthermore, subjects with left main stem (LMS) lesions, proximal left anterior descending (LAD) lesions or triple-vessel involvement were identified.

### Statistical analysis

Statistical analyses were performed using IBM SPSS statistics version 25 (IBM Corporation, Armonk, New York, USA). Categorical variables were presented as percentages and numbers. Continuous variables were presented as mean± SD or as median and interquartile range where appropriate. The patient population was divided into groups of hypertension and no hypertension. The groups were compared using unpaired Student’s *t*-test for continuous variables, Chi-Square test for categorical variables and Mann-Whitney *U* test for variables with skewed distribution. Univariable logistic regression analysis was carried out to explore the association between CV risk factors and extensive non-obstructive CAD. Multivariable logistic regression analysis was used to explore whether hypertension remained independently associated with extensive non-obstructive CAD after adjusting for known covariables. Both uni- and multivariable analyses were reported as odds ratio (OR) with 95% confidence intervals (CI). A two-sided p-value < 0.05 was considered significant.

## Results

### Patient characteristics

Hypertension was present in 45% of the patient population (Table 1). Patients with hypertension were older, with a higher prevalence of obesity, diabetes and statin treatment (all p<0.05, Table 1). Hypertensive patients had lower estimated GFR compared to patients with no hypertension (all p<0.05, Table 1). There was no significant sex difference in the prevalence of hypertension (Table 1). Chest pain was the most common symptom in both groups (Table 1). Clustering of CV risk factors was present in 40% with hypertension compared to 2% without hypertension (p<0.001).

**Table 1:** Clinical and CCTA characteristics of the total population and in subgroups of patients with and without hypertension.

	Total population	Hypertension	No hypertension	P-value
	N = 1138	N = 513 (45%)	N = 625 (55%)	
**Age ±SD, years**	62±11	64±10	61±11	<0.001
**Female, %**	48	51	46	0.170
**BMI ±SD, kg/m** ^**2**^	27.6±4.7	28.6±4.8	26.7±4.4	<0.001
**Obesity, %**	26	34	19	<0.001
**Diabetes mellitus, %**	10	14	7	<0.001
**Smoking, %**	69	66	71	0.073
**Serum creatinin ±SD, μmol/L**	77.9±17.2	79.1±18.8	76.9±15.7	0.033
**Estimated GFR ±SD, mL/min/1.73m** ^**2**^	82.9±15.6	80.4±16.4	84.9±14.7	<0.001
**Chest pain, %**	79	77	81	0.064
**Dyspnea, %**	21	23	19	0.064
**Statin treatment, %**	39	50	29	<0.001
**Calcium score, HU**	40(7–120)	50(10–146)	30(5–97)	0.001
**SIS**	3(3)	3(3)	2(3)	<0.001

Data are mean ±SD, median (IQR; Q1-Q3) or number (%).

BMI: body mass index, CAD: coronary artery disease, GFR: glomerular filtration rate, SIS: segment involvement score, LMS: left main stem, LAD: left anterior descending.

**Fig 1:**
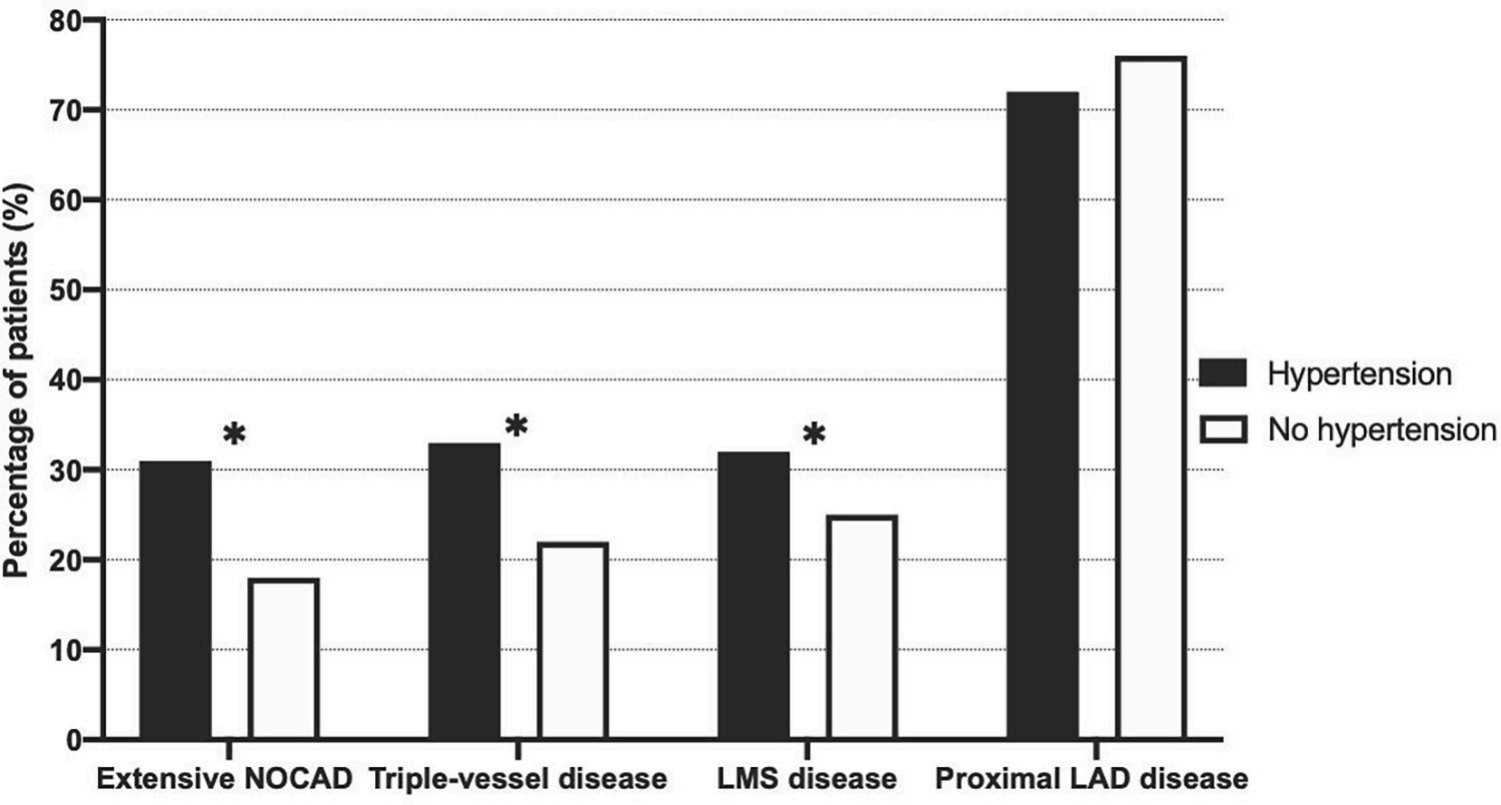
Non-obstructive coronary artery disease characteristics in patients with and without hypertension. NOCAD: non-obstructive coronary artery disease, LMS: left main stem, LAD: left anterior descending. *p <0.05 between groups.

Mean coronary artery SIS was higher in patients with hypertension compared to those without hypertension (p<0.05, Table 1), and extensive non-obstructive CAD was more prevalent among hypertensive individuals (p<0.05, Fig 1). Furthermore, there was a higher prevalence of triple-vessel disease and LMS disease in patients with hypertension compared to patients without hypertension (all p<0.05, Fig 1). Coronary artery calcium score was also significantly higher in patients with hypertension compared to patients without hypertension (p< 0.05, Table 1).

### Covariables of extensive non-obstructive CAD

In univariable logistic regression analysis presence of hypertension, diabetes, statin treatment, as well as higher age, higher calcium score, and female sex were significantly associated with extensive non-obstructive CAD (Table 2). There was no association between extensive non-obstructive CAD and smoking habit or obesity (Table 2). After adjusting for calcium score, statin treatment, sex, age, smoking, diabetes and obesity in multivariable logistic regression analysis, the presence of hypertension in patients with CCS increased the chance of having extensive non-obstructive CAD by 81% (p = 0.004, Table 2), independent of the significant association of higher calcium score (p <0.001), statin treatment (p<0.001) and female sex (p = 0.035).

**Table 2:** Covariables of extensive non-obstructive CAD in univariable and multivariable logistic regression analysis.

	Univariable analysis			Multivariable analysis		
	OR	95% CI	p-value	OR	95% CI	p-value
**Hypertension**	2.07	1.56–2.76	<0.001	1.85	1.22–2.80	0.004
**Calcium score**	1.01	1.01–1.01	<0.001	1.01	1.01–1.01	<0.001
**Statin treatment**	1.85	1.39–2.48	<0.001	1.64	1.09–2.48	0.017
**Sex**	1.36	1.02–1.81	0.037	1.57	1.03–2.40	0.035
**Age**	1.04	1.03–1.06	<0.001	1.02	1.00–1.04	0.059
**Smoking**	1.06	0.75–1.49	0.757	1.13	0.73–1.73	0.591
**Diabetes mellitus**	1.81	1.17–2.78	0.007	1.18	0.63–2.20	0.614
**Obesity**	1.27	0.92–1.74	0.143	1.55	0.98–2.46	0.063

Variables in the model are as follows: hypertension, calcium score, statin treatment, sex, age, smoking, diabetes mellitus, and obesity.

## Discussion

This study demonstrates that in patients with symptomatic CCS and non-obstructive CAD, hypertension is associated with more extensive non-obstructive CAD independent of other known CV risk factors, coronary artery calcium score, sex and statin treatment. In particular, non-obstructive CAD was more often located in the left main stem in hypertensive patients. Furthermore, hypertensive patients with non-obstructive CAD were older with higher coronary artery calcium score, clustering of other CV risk factors and higher prevalence of triple-vessel disease and comorbidities compared to patients without hypertension.

Hypertension is an important CV risk factor associated with increased prevalence and severity of CAD [15, 23]. The pathophysiological relationship between hypertension and atherosclerosis is well accounted for in previous studies [24, 25]. The elevated BP in hypertension induces mechanical stress on the arterial walls causing micro-vascular remodeling with fibromuscular thickening of the intima and media, resulting in luminal narrowing that limits the myocardial perfusion [25–28]. This initiates endothelial damage with accumulation of low-density lipoprotein cholesterol and chronic inflammation in the arterial wall [24, 29]. Consequently, hypertension directly contributes to aggravation and acceleration of coronary artery atherosclerosis. In addition, non-obstructive atherosclerotic lesions are often characterised by large lipid cores and thin fibrous caps, resulting in increased vulnerability to plaque rupture and acute CV events [29].

The clinical syndrome of ischemia with non-obstructive coronary arteries (INOCA) is a common finding in patients undergoing CCTA due to suspected CCS [3]. Myocardial ischemia has previously been demonstrated in hypertension without obstructive coronary arteries and is associated with hypertension mediated target organ damage such as left ventricular hypertrophy and increased aortic stiffness in patients with symptomatic CCS [16, 17, 30]. Previous studies emphasise the importance of coronary abnormalities such as micro-vascular and endothelial dysfunction causing myocardial ischemia in non-obstructive CAD [31–33]. Our study adds to this by demonstrating more extensive non-obstructive CAD by CCTA and increased coronary artery calcification by calcium score in hypertensive patients.

In general, larger extent of non-obstructive CAD is associated with an increased risk of CV events as well as heart failure with preserved ejection fraction [3–5, 7, 28, 34]. As non-obstructive CAD entails mild plaque to more diffuse and moderate atherosclerosis, the atherosclerotic burden rather than the stenosis itself has been identified as an important risk marker in non-obstructive CAD [4, 7, 8, 19, 35, 36]. In particular, Bittencourt et al. demonstrated that larger extent of non-obstructive CAD was associated with a worse prognosis in patients with CCS in both non-obstructive as well as obstructive CAD [7]. Furthermore, more extensive non-obstructive CAD is associated with abnormal fractional flow reserve [37]. In addition, most coronary atherosclerotic precursors of acute coronary syndromes (ACS) are non-obstructive at baseline CCTA [38]. Accordingly, detailed assessment of extent of CAD as performed in this study may be particularly useful in risk stratification in patients with non-obstructive CAD. Furthermore, it has been demonstrated that coronary artery calcification is a significant predictor of long-term risk in patients with hypertension [6]. Our study adds to current knowledge by demonstrating the independent relationship between hypertension and extensive non-obstructive CAD. In individuals with CCS and non-obstructive CAD diagnosing concomitant hypertension may be clinically important and contribute to identifying individuals at particularly high CV risk [6].

Recommendations for risk stratification and treatment in CCS and non-obstructive CAD are still limited. Furthermore, patients with non-obstructive CAD are often underdiagnosed and undertreated [5]. This is in line with the low prevalence of statin treatment (39%) detected in our population of patients with CCS and non-obstructive CAD. Results from the SCOT-HEART trial suggested that the improved prognosis observed in patients with CCS who underwent CCTA may be explained by life style intervention and statin treatment motivated by the improved detection of CAD by CCTA, in particularly in patients with non-obstructive CAD [8]. This is also supported by recent data from Henzel et al. reporting that lifestyle interventions together with optimal medical treatment may reduce disease progression in non-obstructive CAD, compared to optimal medical treatment alone [39]. By demonstrating the independent relationship between hypertension and extensive non-obstructive CAD, our results suggest hypertension as an additional treatment target that may contribute to optimize the management of patients with CCS and non-obstructive CAD. Whether advanced coronary artery plaque analysis assessing plaque vulnerability by CCTA may add to risk stratification in non-obstructive CAD needs to be explored in future studies. Consequently, the benefit from early and aggressive optimal BP control in patients with CCS and non-obstructive CAD should be explored in future randomized follow-up studies [12, 13, 18].

### Study limitations

This is a large observational study including over 1000 patients with non-obstructive CAD defined by CCTA from a clinical registry. The registry only includes patients referred to CCTA after clinical evaluation by experienced cardiologists, and the study population is therefore representative of patients encountered in clinical practice. There are, however, some study limitations to be considered. First, this is a cross-sectional study and therefore no causal relationships can be established. Second, the NORIC registry holds limited information on some patient demographics, such as BP values and antihypertensive medication class, as well as duration and stage of hypertension and diabetes. Therefore, we were unable to evaluate BP control in patients with treated hypertension at the time of the CCTA examination and possible treatment effect on the extent of non-obstructive CAD. Third, only non-obstructive CAD patients with “stable” chest pain and/or dyspnoea, and a low to moderate CV risk, referred to CCTA due to suspected CAD are included in the study. This may introduce a selection bias, and the results cannot be generalized to all patients with CCS [2].

## Conclusions

Hypertension is associated with extensive non-obstructive CAD in patients with symptomatic CCS independent of the significant association of other CV risk factors, sex, calcium score and statin treatment. Our results emphasise the importance of diagnosing hypertension among patients with CCS and non-obstructive CAD. Whether early and aggressive antihypertensive treatment and optimal BP control may delay or reduce disease progression, impact symptoms and improve prognosis in patients with non-obstructive CAD and CCS should be further evaluated in follow-up studies.

## Supporting information

S1 Checklist STROBE checklist. (DOCX)
