# Supplementary material for: Relationship between hypertension and non-obstructive coronary artery disease in chronic coronary syndrome (the NORIC registry)
Source: PLoS One. 2022 Jan 21;17(1):e0262290. doi: 10.1371/journal.pone.0262290 (PMC8782369; doi:10.1371/journal.pone.0262290)
Supplement: S1 Checklist — (DOCX) [file pone.0262290.s001.docx]

**STROBE Statement – Checklist of items that should be included in reports for *cross-sectional studies***

|  | **Item No.** | **STROBE items** | **Location in manuscript where items are reported** |
| --- | --- | --- | --- |
| **Title and abstract** | | | |
|  | 1 | (*a*) Indicate the study’s design with a commonly used term in the title or the abstract  (*b*) Provide in the abstract an informative and balanced summary of what was done and what was found | See title and abstract. |
| **Introduction** | | | |
| Background/rationale | 2 | Explain the scientific background and rationale for the investigation being reported | Introduction, paragraph 1-3. |
| Objectives | 3 | State specific objectives, including any prespecified hypotheses | Introduction, third paragraph. |
| **Methods** | | | |
| Study design | 4 | Present key elements of study design early in the paper | Methods, first paragraph. |
| Setting | 5 | Describe the setting, locations, and relevant dates, including periods of recruitment,  exposure, follow-up, and data collection | Methods, first paragraph, |
| Participants | 6 | (*a*) Give the eligibility criteria, and the sources and methods of selection of  participants | Methods, first paragraph. |
| Variables | 7 | Clearly define all outcomes, exposures, predictors, potential confounders, and effect  modifiers. Give diagnostic criteria, if applicable | Methods, paragraph 2 and 4. |
| Data sources/measurements | 8* | For each variable of interest, give sources of data and details of methods of  assessment (measurement). Describe comparability of assessment methods if there is  more than one group | Methods, paragraph 2 and 4. |
| Bias | 9 | Describe any efforts to address potential sources of bias | Strengths and limitations. |
| Study size | 10 | Explain how the study size was arrived at | Methods, first paragraph. |
| Quantitative variables | 11 | Explain how quantitative variables were handled in the analyses. If applicable,  describe which groupings were chosen and why | Methods, statistical analyses. |
| Statistical methods | 12 | (*a*) Describe all statistical methods, including those used to control for confounding  (*b*) Describe any methods used to examine subgroups and interactions (*c*) Explain how missing data were addressed (*d*) If applicable, describe analytical methods taking account of sampling strategy (*e*) Describe any sensitivity analyses | Methods, statistical analyses. |
| **Results** | | | |
| Participants | 13* | (a) Report numbers of individuals at each stage of study—e.g. numbers potentially eligible, examined for eligibility, confirmed eligible, included in the study, completing follow-up, and analysed (b) Give reasons for non-participation at each stage  (c) Consider use of a flow diagram | Results, table 1. |
| Descriptive data | 14* | (a) Give characteristics of study participants (e.g. demographic, clinical, social) and  information on exposures and potential confounders  (b) Indicate number of participants with missing data for each variable of interest | Results, paragraph 1-2, table 1 and figure 1. |
| Outcome data | 15* | Report numbers of outcome events or summary measures | Results, paragraph 3 and table 2. |
| Main results | 16 | (*a*) Give unadjusted estimates and, if applicable, confounder-adjusted estimates and their precision (eg, 95% confidence interval). Make clear which confounders were adjusted for and why they were included (*b*) Report category boundaries when continuous variables were categorized  (*c*) If relevant, consider translating estimates of relative risk into absolute risk for a  meaningful time period | Results, table 2 and paragraph 3. |
| Other analyses | 17 | Report other analyses done—e.g. analyses of subgroups and interactions, and  sensitivity analyses | Results, table 1. |
| **Discussion** | | | |
| Key results | 18 | Summarise key results with reference to study objectives | Discussion, paragraph 1. |
| Limitations | 19 | Discuss limitations of the study, taking into account sources of potential bias or imprecision. Discuss both direction and magnitude of any potential bias | Discussion, study limitations. |
| Interpretation | 20 | Give a cautious overall interpretation of results considering objectives, limitations, multiplicity of analyses, results from similar studies, and other relevant evidence | Conclusions |
| Generalisability | 21 | Discuss the generalisability (external validity) of the study results | Discussion, study limitations. |
| **Other information** | | | |
| Funding | 22 | Give the source of funding and the role of the funders for the present study and, if applicable, for the original study on which the present article is based | Funding |

*Give information separately for exposed and unexposed groups.
